# Supplementary material for: Hydrogen Bonding Directed Self-Assembly of a Binuclear Ag(I) Metallacycle into a 1D Supramolecular Polymer
Source: Molecules. 2021 Sep 21;26(18):5719. doi: 10.3390/molecules26185719 (PMC8465845; doi:10.3390/molecules26185719)
Supplement: Supplementary file 1 [file molecules-26-05719-s001.zip › molecules-1381165-supplementary.pdf]

Supplementary Materials for

Hydrogen Bonding Directed Self-Assembly of a Binuclear Ag(I) Metallacycle into a 1D Supramolecular Polymer

*Anna Brzechwa-Chodzyńska, Mateusz Gołdyn, Anna Walczak, Jack M. Harrowfield, and Artur R. Stefankiewicz **

* Correspondence: ars@amu.edu.pl

Table of contents

1. Experimental data for the complex $[Pd^{II}L_2](SbF_6)_2$ 2

2. Calculation of void space 5

1. Experimental data for the complex [$Pd^{II}L_2$](SbF_6)$_2$

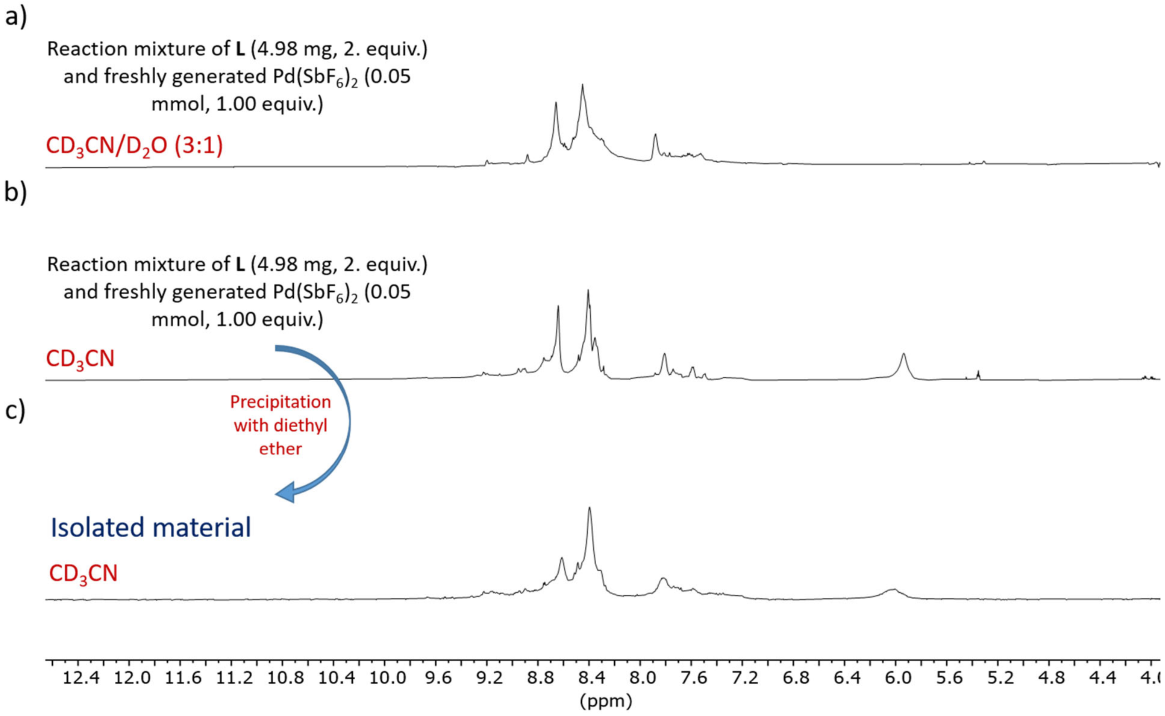

Figure S1. ^{1}H NMR spectrum (600 MHz) of the reaction mixture **[$Pd^{II}L_2$](SbF_6)$_2$** (2.0×10^{-2} M). a) in CD_3CN/D_2O (3:1), b) in CD_3CN, c) isolated material in CD_3CN.

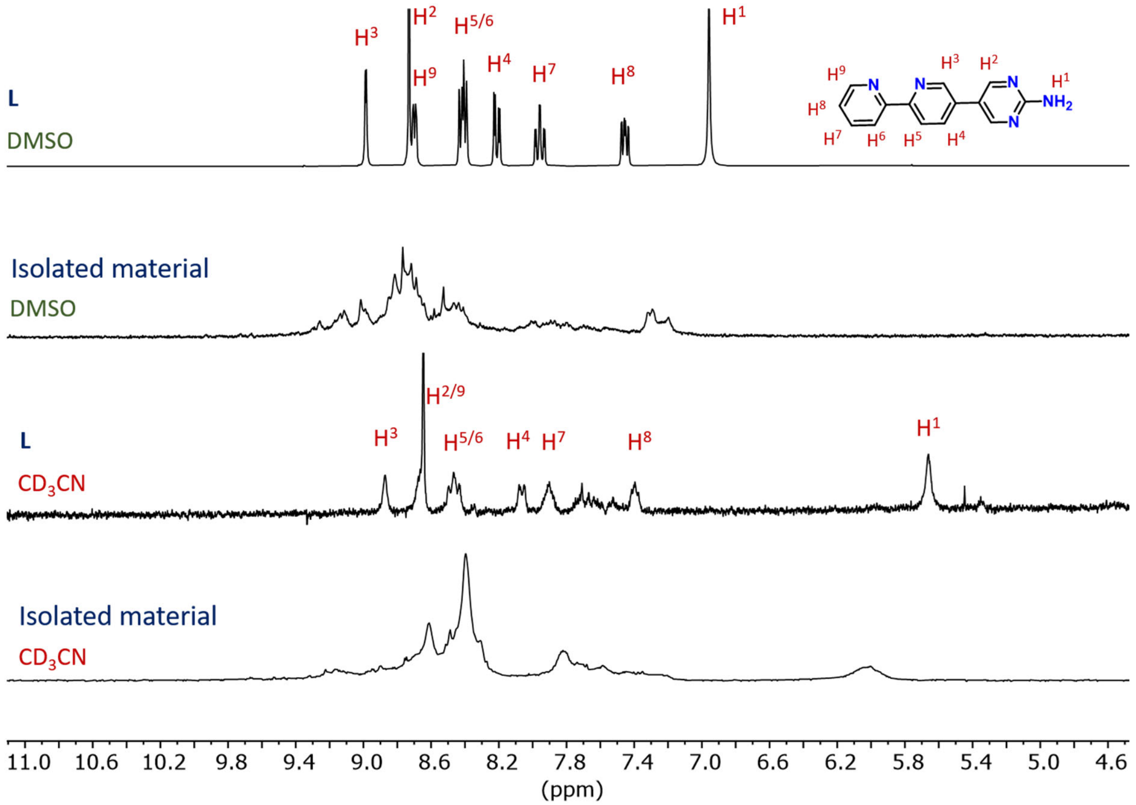

Figure S2. ^{1}H NMR spectrum (600 MHz) of comparisons free ligand and isolated material (see Figure S1.) in deuterated MeCN and DMSO.

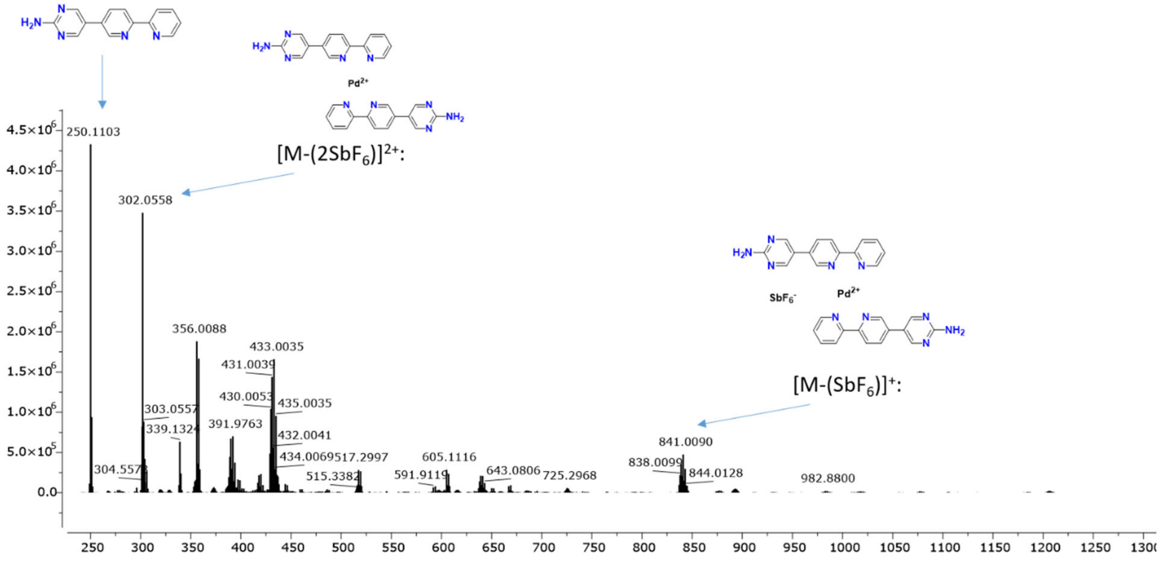

Figure S3. HR-TOF-ESI-MS analysis of the reaction mixture **[Pd^{II}L$_2$](SbF$_6$)$_2$**.

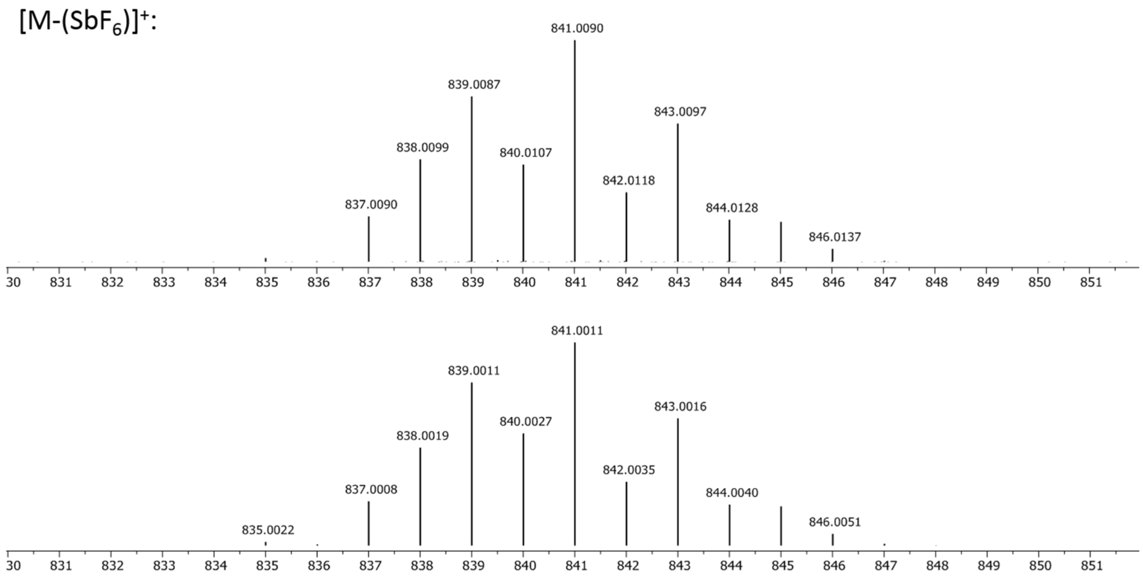

Figure S4. HR-TOF-ESI-MS analysis of the reaction mixture **[Pd^{II}L$_2$](SbF$_6$)$_2$**, showing the observed data (top) and the theoretical isotope model (bottom).

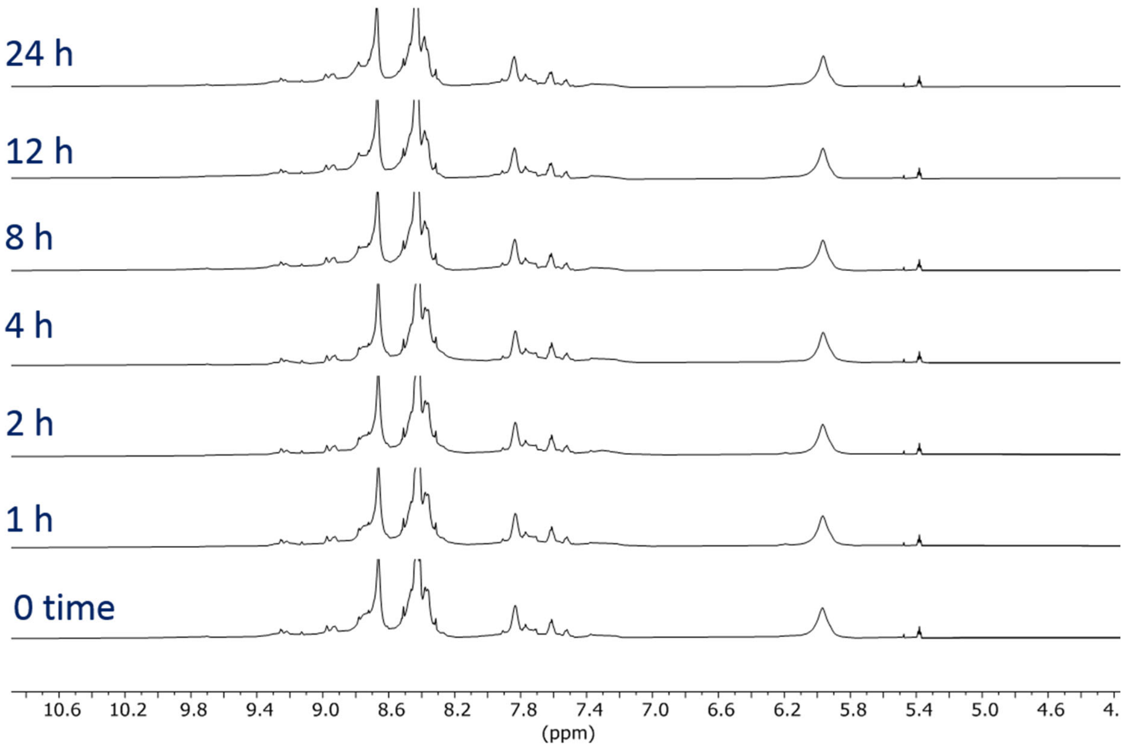

Figure S5. Kinetic ^{1}H NMR spectrum (600 MHz, CD_3CN) of the reaction mixture **[Pd^{II}L$_2$](SbF$_6$)$_2$** (2.0×10^{-2} M). Reaction component: **L** (4.98 mg, 2. equiv.), freshly generated $Pd(SbF_6)_2$ (0.05 mmol, 1.00 equiv.) and 1 mL of acetonitrile-d3.

2. Calculation of void space

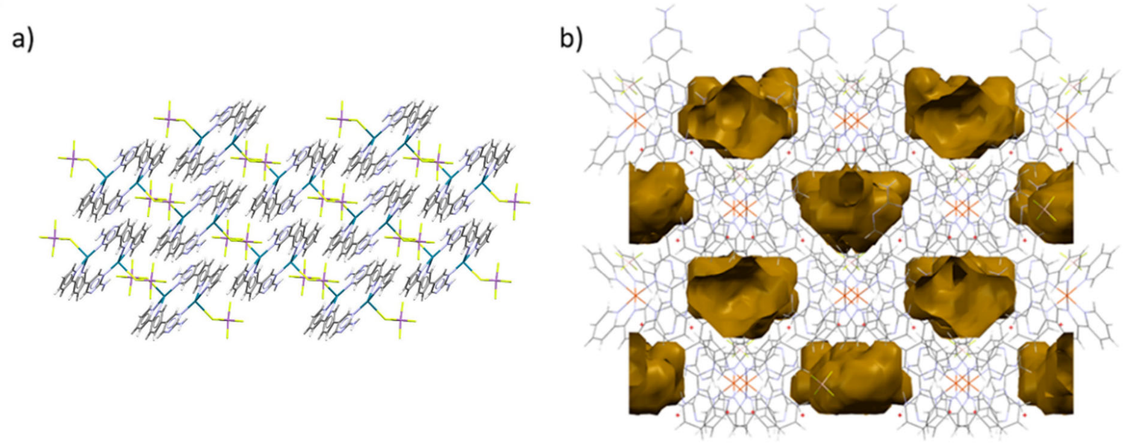

Voids Volume	0.0 % of unit cell volume
	0.00 Å^3

Voids Volume	12.8 % of unit cell volume
	2462 Å^3

Figure S6. Void analysis of a) complex **$[Ag_2L_2](SbF_6)_2$**, b) complex **$[Fe^{II}L_3](BF_4)_2{\times}9H_2O$** performed with MERCURY using a probe radius 1.2 Å and grid spacing of 0.2 Å, calculated using the contact surface algorithm.
